# Supplementary material for: Patterns of Intron Gain and Loss in Fungi
Source: PLoS Biol. 2004 Nov 30;2(12):e422. doi: 10.1371/journal.pbio.0020422 (PMC532390; doi:10.1371/journal.pbio.0020422)
Supplement: Table S1 — Also available at http://genes.mit.edu/NielsenEtAl/. (4.3 MB ZIP). [file pbio.0020422.st001.zip › NielsenEtAl/html/279.html]

AN1993.1.NCU08411.1.MG06530.1.FG10746.1


```
 CLUSTAL W (1.82) Multiple Sequence Alignments - Introns Inserted


Sequence 1: NCU08411.1	429 aa
Sequence 2: FG10746.1	424 aa
Sequence 3: AN1993.1	429 aa
Sequence 4: MG06530.1	428 aa
Alignment Length: 433 aa
Number Identitical Residues: 273 aa
Alignment Score (without introns) 12295


MG06530.1 	MLSTMRIASRQVAGRRMAVR---PLAALRPASTWANVQMGPP0D0AILG1ITEAFKADSN
NCU08411.1	MLSSVRLVTRRAAVRSAFASPGRSLLAVRAASFWANVPQGPP0D0AILG1ITEAFKADTF
FG10746.1 	MLSTLRVASRRAARPAQYN-----LAAIRAASNWANVPQGPP0D0AILG1ITEAFKADKF
AN1993.1  	MLSTLKVASRQAASRNASVR--SVFAGARCASTWSNVPQGPP0D~AILG~ITEAYKADSF
          	***::::.:*:.*           : . * ** *:**  *** * **** ****:***. 

MG06530.1 	NKKINLG1VGAYR~DDAGKPYVLPSVRQAEEKVIAARMNKEYAGITGVPEFTSGALTLAY
NCU08411.1	DKKINLG1VGAYR~DDKGKPYVLPSVRKAEDKVIHSRMNKEYAGITGVPEFTKAAAVLAY
FG10746.1 	DKKINLG1VGAYR~DDAGKPYVLPSVREAEMKVVESKLNKEYAGITGVPEFPPAAAKLAY
AN1993.1  	KEKINLG~VGAYR1DDQGKPYVLPSVRAAEDKVVASRFDKEYAGITGIPSFTKAAAQLAY
          	.:***** ***** ** ********** ** **: ::::********:*.*. .*  ***

MG06530.1 	GKDSTAL--DRTAITQSISGTGALRIGAEFLSKFYPGAKNIYIPTPSWANHGAVFTQAGL
NCU08411.1	GKDSSAL--DRLVITQSISGTGALRIGGEFLNRFFPGAKKIYIPTPSWANHGAVFRDSGL
FG10746.1 	GANSPAL--DRITITQTISGTGALRVGAAFLAKFFPGEKKIYIPQPSWANHKAVFNHAGL
AN1993.1  	GADSPVLKEDRLVITQSISGTGALRIGGAFLQRFYPGAKKIYLPTPSWANHNAVFKDSGL
          	* :*..*..** .***:********:*. ** :*:** *:**:* ****** *** .:**

MG06530.1 	KVEKYRYYNKDTIGLDFEGLVADVKAAPKGSIFLFHACAHNPTGVDPTPEQWREIETVVK
NCU08411.1	EVAQYAYYDKNTIGLDFEGLIRDLKAAPNSSIFLFHACAHNPTGVDPTPEQWKEIEAVVK
FG10746.1 	EVEQYRYYDKKTIGLDFEGLIADVKSAPNGSVFLFHACAHNPTGVDPTQEQWKQISDVVK
AN1993.1  	EVEKYRYYNKDTIGLDFEGLVEDLKAAPNNSIILLHACAHNPTGVDPTQDQWRQISNVMK
          	:* :* **:*.*********: *:*:**:.*::*:************* :**::*. *:*

MG06530.1 	SAGHYAFFDMAYQGFASGDIHKDAFALRHFVAQGHNVCLAQSFAKNM~GLYGERVGAFSI
NCU08411.1	DKGHYSFFDMAYQGFASGDIHKDAFAVRYFVEQGHNICLAQSFAKNM~GLYGERVGAFSI
FG10746.1 	EKGHFAFFDMAYQGFASGDTDKDAFAVRYFVEQGHNIALCQSFAKNM0GLYGERIGAFSM
AN1993.1  	EKGHFAFFDMAYQGFASGNADTDAFAPRHFVEQGHNIALCQSFAKNM0GLYGERVGAFSL
          	. **::************: ..**** *:** ****:.*.******* ******:****:

MG06530.1 	VCDSAEEKKRVDSQLKILVRPMYSNPPIHGARIAAQILNTPALYDQWLAEVKGMADRIIT
NCU08411.1	VCADADEKKRVDSQVKILVRPLYSNPPIHGARIAAEILNTPELYEQWLTEVKEMADRIIK
FG10746.1 	VCADADEKKRVDSQLKILIRPLYSNPPIHGARIAAEILNSPTLYKQWLGEVKEMADRIIT
AN1993.1  	VCESAEEKKRVDSQIKILIRPFYSNPPVHGARVASTIMNDPALNKQWLGEVKGMADRIIE
          	** .*:********:***:**:*****:****:*: *:* * * .*** *** ****** 

MG06530.1 	MRALLKENLEKLGSKHDWSHITSQ~IGMFAYTGLTPEQMEQLAKS~HSVYATKDGRIRSL
NCU08411.1	MRALLKENLEKLGSKHDWSHITSQ~IGMFAYTGLTPEQMEKLAKE~HSVYATKDGRIS--
FG10746.1 	MRALLKDNLEKLGSKHDWSHITSQ~IGMFAYTGLTSEEMTRLAEE~FSVYATKDGRIS--
AN1993.1  	MRALLKKNLEELGSKHDWTHITSQ0IGMFAYTGLKPEQMEALAKE0HSVYATKDGRIS--
          	******.***:*******:***** *********..*:*  **:. .**********   

MG06530.1 	VSPARTLAVLLRLSSRSRVRW
NCU08411.1	VAGITSENVGRLAEAIFKVKG
FG10746.1 	VAGITSENVGRLAEAIYKVKG
AN1993.1  	VAGITTGNVKRLAESIYKVTG
          	*:   :  *    .:  :*
```
